# Comparison of the Proteomes and Phosphoproteomes of *S. cerevisiae* Cells Harvested with Different Strategies

**DOI:** 10.3390/proteomes11040028

**Published:** 2023-09-27

**Authors:** Valentina Rossio, Joao A. Paulo

**Affiliations:** Department of Cell Biology, Harvard Medical School, Boston, MA 02115, USA

**Keywords:** TMTpro, yeast, proteome analysis, phosphoproteome analysis, stress

## Abstract

The budding yeast *Saccharomyces cerevisiae* is a powerful model system that is widely used to investigate many cellular processes. The harvesting of yeast cells is the first step in almost every experimental procedure. Here, yeast cells are isolated from their growth medium, collected, and used for successive experiments or analysis. The two most common methods to harvest *S. cerevisiae* are centrifugation and filtration. Understanding if and how centrifugation and filtration affect yeast physiology is essential with respect to downstream data interpretation. Here, we profile and compare the proteomes and the phosphoproteomes, using isobaric label-based quantitative mass spectrometry, of three common methods used to harvest *S. cerevisiae* cells: low-speed centrifugation, high-speed centrifugation, and filtration. Our data suggest that, while the proteome was stable across the tested conditions, hundreds of phosphorylation events were different between centrifugation and filtration. Our analysis shows that, under our experimental conditions, filtration may cause both cell wall and osmotic stress at higher levels compared to centrifugation, implying harvesting-method-specific stresses. Thus, considering that the basal activation levels of specific stresses may differ under certain harvesting conditions is an important, but often overlooked, aspect of experimental design.

## 1. Introduction

The budding yeast *S. cerevisiae* is a broadly used model system to study the cellular processes that are conserved throughout the evolution [1]. The first step in many experimental procedures involving *S. cerevisiae*, as well as other model systems, is cell harvesting, which isolates the cells from their growth medium. Often, this step is considered trivial, and the details are frequently overlooked, yet its importance may be critical for downstream data interpretation. Understanding if cell harvesting can activate specific stress responses is important to address as it could affect the results of the experiments and their downstream interpretation.

Centrifugation and filtration are the two most common methods used to harvest *S. cerevisiae* cells. During both centrifugation and filtration, cells are subjected to external forces that they do not usually experience in their natural environment. During centrifugation, cells are subjected to higher centrifugal force, while during filtration they experience unusually high pressure. It is possible that the thick cell wall protects yeast cells from stresses during these procedures, but if and how yeast cells respond to these commonly used harvesting methods has, to date, not been clearly investigated. 

Post-translational modifications, specifically phosphorylation, are strategies employed by cells and unicellular organisms to quickly respond and/or adapt to changes in their environment. Phosphorylation events are highly specific, reversible, and dynamic, which highlights their importance in signaling mechanisms. Moreover, the different proteoforms generated by phosphorylation, like other post-translational modifications, also increase the complexity of the proteome. A large network of protein kinases (over 100 in *S. cerevisiae*) drive the phosphorylation reactions by recognizing specific consensus sequences on their substrates. Mass-spectrometry (MS)-based phosphoproteomics is a powerful method to globally identify phosphorylation events. In particular, isobaric label-based (TMTpro) quantitative mass spectrometry facilitates the identification and quantification of thousands of phosphorylation events in a single experiment across multiple conditions. Enriching and analyzing the phosphopeptides detected in global proteomic experiments facilitates the analysis of the activity of specific protein kinases and pinpoints which of these are involved in particular biological processes. 

Here, we compared the proteome and the phosphoproteome of *S. cerevisiae* wild type cells after harvesting them with three different, but commonly employed, methods: low-speed centrifugation, high-speed centrifugation, and filtration. We used isobaric label-based (TMTpro) quantitative mass spectrometry to profile the proteomes and phosphoproteomes as a means to investigate the effects of these cell harvesting strategies. We found that the proteome was stable across the tested conditions, as may be expected in the time scale of several minutes. However, hundreds of phosphorylation events were altered between the filtration and centrifugation harvesting strategies. Our analysis revealed specific kinases that are activated or inhibited during the conditions tested and that can be, in part, responsible for the changes in the phosphorylation events that we measured. Our analysis suggests that filtration may increase cell wall stress and cause a sudden change in osmolarity that does not occur during centrifugation. This finding suggests that different harvesting methods can induce specific stresses on a short time scale and highlights the importance of taking into consideration the fact that the basal activation levels of these stresses may be different under certain harvesting conditions. 

## 2. Material and Methods

### 2.1. Materials

The *S. cerevisiae* strain used in this study is a wild type strain with a BY4742 genetic background (*MATalpha*, *his3Δ1*, *leu2Δ0*, *lys2Δ0*, *ura3Δ0*). It is commercially available and was purchased at Horizon Scientific (Cambridge, UK). YPD (yeast extract, peptone, dextrose) media was from Sunrise Science (Knoxville, TN, USA). Tandem Mass Tag (TMTpro) isobaric tagging reagents, BCA (Bicinchoninic acid) protein quantification kit, Pierce protease inhibitor tablets, trypsin protease, and Pierce C18 tips were purchased from ThermoFisher Scientific (Rockford, IL, USA). C18 StageTip—(Empore) material was purchased from CDSanalytical (Oxford, PA, USA). Sep-Pak cartridges (100 mg) were acquired from Waters (Milford, MA, USA). Lys-C protease was from Fujifilm Wako (Richmond, VA, USA). Mass spectrometry grade reagents (i.e., water, formic acid, methanol, and acetonitrile) were purchased from J.T. Baker (Center Valley, PA, USA). 

### 2.2. Yeast Growth and Protein Extraction

*S. cerevisiae* cultures were grown overnight in triplicate at 30°C in YPD medium [2]. The next morning, triplicate cultures were diluted with fresh YPD medium to OD_600_ = 0.15 and grown until OD_600_ = 0.8 (exponential phase). Successively, 50 mL of culture of each sample were processed. Cells were harvested by low-speed centrifugation (400 rpm (20× *g*) for 5 min), high-speed centrifugation (4000 rpm (1500× *g*) for 5 min), or filtration (less than 5 min). A nylon filter of pore size 0.45 μm (Agilent Nylon 0.66, part no. R000038114) was used to filter the cells. In all cases, cells were washed twice with 25 mL of cold sterile water. For the centrifugation method, both washes, of five minutes each, were executed at the same speed as the centrifugation to collect the cells. For the filtration strategy, cells were washed twice by filtration. Following the washing, the filters with the cells were removed and placed in a conical tube. All the samples were then flash frozen and stored at −80 °C until sample processing. Cell lysis, protein extraction, and reduction were executed as described previously [3]. Briefly, cell pellets were resuspended in lysis buffer (8 M urea, 200 mM EPPS (4-(2-hydroxyethyl)-1-piperazinepropanesulfonic acid), pH 8.5 containing protease inhibitors) and lysed by bead-beating in the cold room. Protein concentrations were determined using a BCA assay performed according to the manufacturer’s instructions. Proteins were reduced through treatment with 5 mM tris(2-carboxyethyl) phosphine (TCEP) for 20 min, alkylated with 10 mM iodoacetamide for 20 min, and lastly quenched with 10 mM dithiothreitol (DTT) for 20 min. Alkylation and quenching were performed in the dark and all the reactions were incubated at room temperature. A total of 100 µg of protein from each sample was precipitated using chloroform–methanol precipitation [3]. Briefly, we added 400 µL of 100% methanol to each sample and vortexed for 5 sec. We then added 100 µL of 100% chloroform and vortexed once again. Finally, we added 300 µL of water, vortexed, and centrifuged for 5 min at 15,000 g. After centrifugation, two layers are formed (one aqueous and one organic) with a white protein disk between them. We aspirated both layers by gently tilting the tube ~45 degrees, thereby leaving the disk intact. We washed the protein pellet once with 800 µL methanol. We then vortexed briefly and centrifuged at 15,000× *g* for 2 min. We removed the supernatant without drying the pellet completely and resuspended the samples in 100 µL of 200 mM EPPS (pH 8.5) for enzymatic digestion.

### 2.3. Protein Digestion, TMT Labeling and Sample Processing

Samples were digested using Lys-C (overnight at 24 °C) and trypsin (6 h at 37 °C). An amount of 1 µg of each enzyme was used per 100 µg of protein. A final volume of 30% acetonitrile was added to each digest followed by the addition of specified tandem mass tag (TMTpro) labeling reagents. A total of 50 µg of peptide for each sample was labeled with 100 µg of TMTpro reagents as follows: low-speed triplicates: 126, 127n, 127c; high- speed triplicates: 128n, 128c, 129n; filtration triplicates:129c, 130n, 130c. Samples were incubated for one hour at room temperature. 

Prior to proceeding with the final pooling of the samples and fractionation, we performed a quality control step in which a small amount of each sample was pooled and analyzed using mass spectrometry to confirm successful peptide digestion, the degree of labeling, and equal amount of protein per channel. We combined ~1 µg of peptide from each sample, mixed, and desalted via StageTip [4] to verify labeling efficiency (ensuring that it is >97%). For desalting, we used the equivalent of 6 disks per StageTip. Our C18 disks were 0.4 mm in diameter and 0.5 mm in length, each having a binding capacity of 2–4 µg of digested proteins. StageTips were made manually by inserting these small disks of C18 beads embedded in a soft mesh of PTFE (Polytetrafluoroethylene) into a P200 pipette tip.

Upon verifying the labeling efficiency (>97%), the labeling reactions were quenched through the addition of hydroxylamine to a final concentration of ~0.3% (15 min at room temperature). Samples were combined 1:1 to ensure that each channel contained the same amount of peptide. The pooled peptide sample was desalted with a 100 mg Sep-Pak solid phase extraction column. The sample was now subjected to spin column-based phosphopeptide enrichment, as described below. The unbound peptides were then fractionated with basic pH reversed-phase (BPRP) HPLC using an Agilent 1200 pump with an Agilent 300Extend C18 column (2.1 mm inner diameter, 3.5 μm particles, and 250 mm in length). A 50 min linear gradient of 5% to 35% acetonitrile in 10 mM ammonium bicarbonate pH 8 was used with a flow rate of 0.25 mL/min. In all, 96 fractions were collected and then concatenated to 24 superfractions [5]. These 24 superfractions were divided into two non-adjacent sets of 12 superfractions that were acidified with formic acid to 1% (final concentration). Lastly, these fractions were vacuum centrifuged to near dryness, and each was desalted via StageTip, dried by vacuum centrifugation, and reconstituted in 5% acetonitrile and 5% formic acid prior to LC-MS/MS analysis.

### 2.4. Spin Column-Based Phosphopeptide Enrichment

For phosphopeptide enrichment, the High-Select Fe-NTA Phosphopeptide Enrichment Kit was used [6]. The only deviation from the manufacturer’s protocol was that the eluates were collected in a pre-prepared “elution collection tube” with 100 µL of 10% formic acid. The combined eluate was vacuum centrifuged to near dryness and desalted via StageTip [4] prior to mass spectrometry analysis.

### 2.5. Mass Spectrometry Data Acquisition and Processing

Mass spectrometric data were collected on an Orbitrap Fusion Lumos mass spectrometer that is in line with a Proxeon NanoLC-1200 UHPLC and a FAIMSpro interface [7]. For peptide and phosphopeptide fractionation, we pulled and packed a 100 μm capillary column in the lab with 35 cm of Accucore150 resin (150 Å, 2.6 μm; ThermoFisher Scientific). 

For whole proteome profiling, the scan sequence began with an MS1 spectrum that was collected in the Orbitrap mass analysis. Our settings included: resolution of 60,000, scan range of 350–1350 Th, automatic gain control (AGC) target of 100%, and the maximum injection time was set as “auto.” MS2 data acquisition in the Orbitrap consisted of higher-energy collisional dissociation (HCD). Our settings included AGC of 200%, maximum injection time: 86 ms, NCE (normalized collision energy) of 36%, and an isolation window of 0.7 Th. In all, 24 RAW files were collected. Data for 12 non-adjacent superfractions acquired using a compensation voltage (CV) set of −40/−60/−80 V, while that for the other 12 superfractions were acquired with a CV set of −30/−50/−70 V. A 1 sec TopSpeed cycle was used for each CV and data were collected over a 90 min gradient. 

For phosphopeptide profiling, data were acquired with either an Orbitrap Fusion Lumos mass spectrometer that is coupled to a Proxeon NanoLC-1200 UHPLC (as above) or an Orbitrap Eclipse mass spectrometer that is coupled to a Vanquish Neo UHPLC. Both systems used a similar column to that described above, and a FAIMSpro interface [4]. For the Lumos, the scan sequence began with an Orbitrap MS1 spectrum with the following parameters: resolution: 60,000, scan range: 350–1400 Th AGC: 100%, and maximum injection time: 50 ms. The subsequent Orbitrap-based MS2 analysis consisted of HCD with the following parameters: resolution: 50,000, AGC: 300%, NCE: 36%, maximum injection time: 250 ms, and isolation window: 0.7 Th. We also excluded unassigned, singly, and >5+ charged species from MS2 analysis and we set the dynamic exclusion to 60 s so as not to fragment the same precursor multiple times. Three RAW files were collected for phosphorylation profiling using a compensation voltage (CV) sets of −40 V, −60 V, −80 V and −30 V, −50 V, −70 V on the Lumos and −40 V, −60 V, −80 V on the Eclipse. A TopSpeed cycle of 1 s was used for each CV on both instruments.

Spectra were converted to mzXML using MSconvert [8]. Database searching included all *S. cerevisiae* entries from UniProt (downloaded July 2022) as well as all protein sequences for that database in the reversed amino acid sequence order. Searches were performed using a 50 ppm precursor ion tolerance for total protein level profiling and a product ion tolerance of 0.9 Da to maximize sensitivity in conjunction with Comet database searching and linear discriminant analysis (LDA) [9,10]. TMT tags on peptide N termini and lysine residues (+304.207 Da), as well as carbamidomethylation of cysteine residues (+57.021 Da), were set as static, while oxidation of methionine (+15.995 Da) was set as variable. For phosphopeptide analysis, +79.966 Da was set as a variable modification on serines, threonines, and tyrosines. Peptide-spectrum matches (PSMs) were adjusted to a 1% false discovery rate (FDR) [11,12], and filtering thereof was performed using LDA [10] and assembled to a final protein-level FDR of 1% [12]. Moreover, for phosphorylation analysis, site localization was determined using AScore [9]. Proteins were quantified by summing reporter ion counts across PSMs [13]. Reporter ion intensities were adjusted to correct for isotopic impurities according to the manufacturer’s insert. The signal-to-noise (S/N) measurements of peptides assigned to each protein were summed and normalized so that the sum of the signal for all proteins in each channel was equal, thereby accounting for equal protein loading (i.e., column normalized). Finally, each protein abundance measurement was scaled, such that the summed S/N for that protein across all channels equaled 100, to generate a relative abundance (RA) measurement. 

## 3. Results and Discussion

### 3.1. Proteome-Wide Abundance Profiling Revealed Minimal Changes among the Different Harvesting Methods

**Figure 1:**
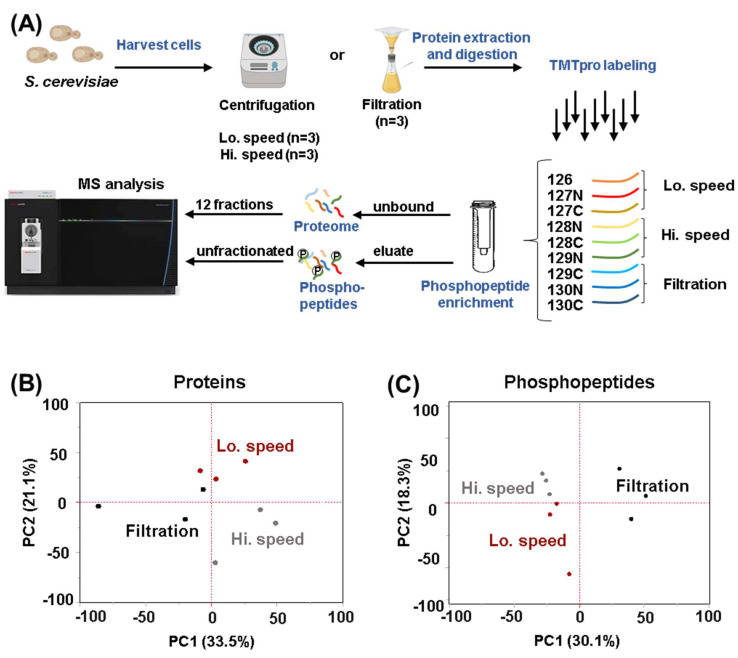
Experimental workflow and principal component analysis (PCA). (**A**) Wild-type yeast cells were grown to exponential phase and harvested in triplicate by three different methods: centrifugation at low-speed (Lo; 400 rpm), centrifugation at high-speed (Hi; 4000 rpm) or filtration. Cells were lysed and proteins were precipitated. Following digestion with LysC and trypsin, peptides were labeled with tandem mass tag (TMT) reagents and pooled 1:1. The peptides were subjected to phosphopeptide enrichment. The eluate (phosphopeptides) and the unbound peptides (whole proteome) were processed separately. The unbound portion of the sample was fractionated using basic pH reversed-phase (BPRP) HPLC. These fractions and the enriched phosphopeptides were analyzed using mass spectrometry (MS). This panel has been assembled, in part, using Biorender.com. PCA diagram of (**B**) the protein and (**C**) the phosphopeptide datasets.

We compared the effects on the proteome and the phosphoproteome in wild type *S. cerevisiae* cells that were harvested with three commonly used methods: low-speed centrifugation (400 rpm), high-speed centrifugation (4000 rpm), and filtration (Figure 1A). We quantified 4631 proteins and 3331 phosphopeptides across all the conditions. All proteins, peptides, and their associated relative abundances used for quantitation are reported in Table S1, while the equivalent for phosphopeptides are reported in Table S2. 

**Figure 2:**
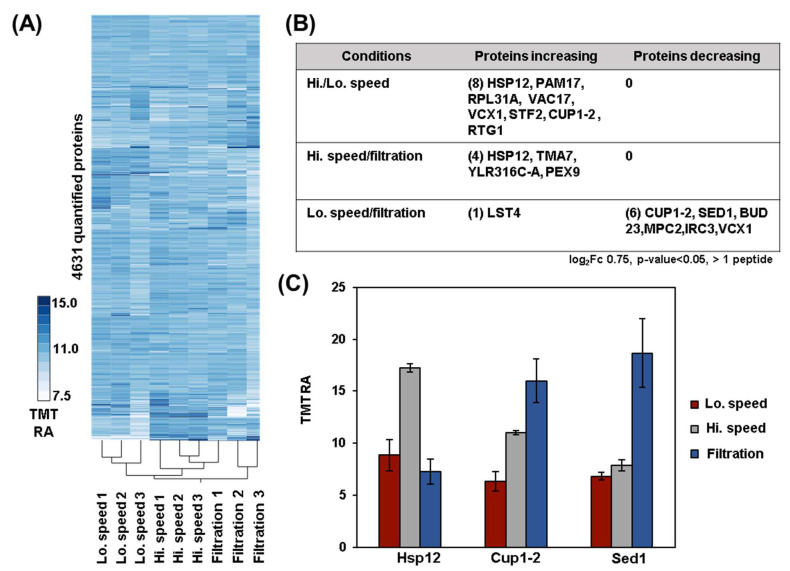
Global proteome analysis and exploration of stress-induced proteins differentially regulated across the tested conditions. (**A**) Hierarchical clustering analysis of the TMT relative abundance (TMT RA) for proteins quantified across the 9 TMT channels. (**B**) Table summarizing the number of differentially abundant proteins quantified under the tested conditions. Selection thresholds are indicated below the table. (**C**) The highlighted proteins are known to be stress-induced. These include the heat shock protein Hsp12; the copper-activated metallothionine Cup1–2 and the stress-induced GPI-cell wall glycoprotein, Sed1. Error bars: Standard deviation of replicates.

We first performed a principal component analysis (PCA) for both the protein and phosphopeptide datasets (Figure 1B,C). PCA showed clustering of the replicates based on the tested conditions for both proteins and phosphopeptides. In fact, the first two principal components (PC1, PC2) together accounted for 54.6% of the variance for the protein dataset (Figure 1B) and for the 48.4% of the variance for the phosphopeptide dataset (Figure 1C). Next, we analyzed the proteome by performing hierarchical clustering using Euclidean distance with Ward’s inter-cluster linkage (Figure 2A). We noted imperfect clustering among the replicates, which is most likely due to the very few significantly changing proteins among the three conditions. As such, slight variability in sample preparation and/or instrumental noise drives the clustering more than differences in the proteome. Overall, we observed the anticipated strong proteome stability over the short time of harvesting (~5 min) as the abundances of very few proteins were altered under the harvesting methods tested. Specifically, we detected only eight differentially abundant proteins (log_2_ fold-change +/− 0.75, *p*-value < 0.05) between high- and low-speed centrifugation, four between high-speed centrifugation and filtration, and seven between low-speed centrifugation and filtration (Figure 2B). Interestingly, some of these differentially abundant proteins were known to be stress-induced. For example, the protein abundance of the heat shock factor Hsp12, that regulates membranes organization, were higher after centrifugation at high speed (Figure 2C) compared to the other two harvesting methods. This finding suggested that high-speed centrifugation can induce more membrane stress than the two other harvesting methods. In contrast, the protein abundance of the major copper-activated metallothionine Cup1–2 and the major stress-induced structural GPI-cell wall glycoprotein Sed1 are both higher after filtration compared to centrifugation (Figure 2C). 

### 3.2. The Quantification of Hundreds of Phosphorylation Events Differed between Cells Harvested by Filtration and Centrifugation

**Figure 3:**
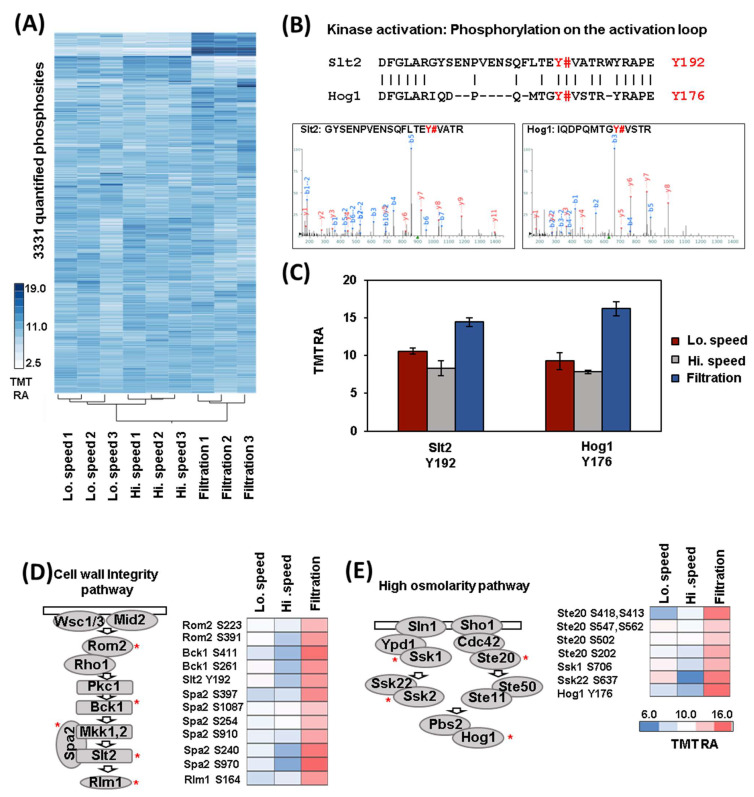
Hierarchical clustering analysis and example of kinases that are differentially regulated through phosphorylation of their activation loop. (**A**) Hierarchical clustering analysis of the TMT relative abundance (TMT RA) measurements for phosphorylation sites quantified across the 9 TMT channels. (**B**) Top: Amino acid alignment of the activation loop of Hog1 and Slt2 kinases. Phosphorylation sites that activate these two kinases are highlighted in red. Bottom: Spectra of the phosphopeptides detected by mass spectrometry are shown. (**C**) The TMT RA measurements of the phosphorylation sites across the different conditions tested. Error bars: Standard deviation of replicates. (**D**) The TMT RA values of phosphorylation sites are presented for several members of the cell wall integrity pathway (left) and (**E**) the high osmolarity pathway and they are labeled with a red asterisk in the figure.

As we carried out for the proteome, we first performed hierarchical clustering using the values of the 3331 phosphorylation events (Figure 3A). The samples clustered, as expected, as the triplicates of each harvesting method grouped together. While only 31 phosphorylation events were altered (log_2_ fold-change +/− 0.75, *p*-value < 0.05) between high- and low-speed centrifugation, hundreds changed when comparing filtration with centrifugation. In particular, 275 phosphorylation events were altered between high-speed centrifugation and filtration, while 145 between low-speed centrifugation and filtration (Figure S1A). This finding was significant as the proteome was mostly unchanged over this short time span. Next, we used PhosphoSitePlus motif analysis [14] to pinpoint the consensus amino acid sequences of the phosphorylation events that were significantly altered between high-speed centrifugation and filtration. This analysis revealed that the phosphorylation events that changed included motifs with the sequences: RRXSP (increasing) and RXXSP (decreasing). Both of these amino acid motifs were consensus sites for the CMGC kinases family (Figure S1B), which includes proline-directed kinases that are involved in many cellular signal transduction pathways [15].

### 3.3. Analysis of Differentially Regulated Kinases through Phosphorylation

As hundreds of phosphorylation events differed between filtration and centrifugation, we hypothesized that some kinases could be differentially regulated between these two conditions. Consistent with this, we found multiple phosphorylation events (n = 17) on protein kinases (n = 13) and phosphatases (n = 1) (Figure S1C) that increase after filtration compared to centrifugation. In particular, multiple sites belong to different kinases involved in the cell wall integrity pathway, such as Bck1, Slt2, and Stt4, and to the high osmolarity pathway, such as Hog1 and Ssk22. A detailed analysis revealed that phosphorylation in the activation loop of both the mitogen activated kinases Slt2 (Mpk1) and Hog1 increased more after filtration compared to either centrifugation-based harvesting method (Figure 3B,C). Slt2 is the central regulator of the cell wall integrity pathway which ensures the maintenance of cell wall integrity [16]. The Hog1 (high osmolarity glycerol 1) pathway, of which the Hog1 kinase is the main player, is activated in the presence of increased external osmolarity. This finding suggests that filtration may increase cell wall stress and cause a sudden change in osmolarity that does not occur during centrifugation. Both these kinases belong to the CMGC family, supporting our previous consensus site analysis (Figure S2B). Consistent with the activation of Mpk1 and Hog1, we also found that multiple phosphorylation events for other key components of the cell wall integrity (Figure 3D) and osmolarity (Figure 3E) pathways increased after filtration compared to centrifugation. These phosphorylations are likely a consequence of the activation of these two pathways. For the cell wall integrity pathway, we observed increased phosphorylation for the guanine nucleotide exchange factor of Rho1, Rom2 for the mitogen-activated protein MAP kinase kinase kinase (MAPKKK), Bck1 for the scaffold protein of Mkk1p and Mpk1, Spa2 and for the transcription factor, Rlm1 (Figure 3D). Among the components of the osmolarity pathway, we observed increased phosphorylation for the kinase Ste20, for the cytoplasmic intermediate osmosensor, Ssk1, and for the MAPKKK, Ssk22 (Figure 3E). Kinase activity can also be regulated by phosphorylation on residues outside the activation loop [15]. One example of such regulation is the main cell cycle kinase Cdk1, Cdc28, in which phosphorylation on Tyrosine-19 (Tyr19) (outside of the activation loop) is inhibitory [17]. This phosphorylation event was relatively higher after filtration than after centrifugation (Figure S1D). The phosphorylation of Cdc28 on Tyr19 is regulated by the Wee1-related kinase Swe1 and the Cdc25-related phosphatase Mih1 and blocks the cell cycle by impeding entry into mitosis (Figure S1D) [17]. The phosphorylation of Cdc28 on Tyr19 is a central event of the morphogenesis checkpoint that halts mitotic entry when complications arise in the budding process [18]. This checkpoint can be activated by environmental stresses that induce a temporary depolarization of the actin cytoskeleton or affect bud formation. Both the kinases Hog1 and Mpk1 have been shown to activate the morphogenesis checkpoint [19,20]. Our data may suggest that the increase in phosphorylation of Tyr19, that we observed after filtration, could be a result of the activation of one or both kinases. Thus, our analysis revealed that filtration can induce specific stress pathways that differ from those related to centrifugation, as reflected in the difference in phosphorylation events among the strategies investigated. 

## 4. Conclusions and Limitations

Here, we presented a quantitative, global proteome, and phosphoproteome analysis of wild type *S. cerevisiae* cells harvested with three commonly used methods: low-speed centrifugation, high-speed centrifugation, and filtration. We profiled over 4600 proteins which were mostly unaltered under the different conditions. We also profiled approximately 3300 phosphorylation events and we found hundreds that were altered when comparing centrifugation and filtration. We have provided a list of proteins and phosphorylation events that may be altered due to stress response when cells are harvested by centrifugation or filtration. These lists could serve as a starting point for those interested in such proteins or be used to support other previously collected data concerning the highlighted proteins and phosphorylation events, which can be further supported by orthogonal and more targeted experiments. We pinpointed specific kinases that are differentially regulated by phosphorylation during the tested conditions, such as the MAP kinases Hog1 and Mpk1 and the main cell cycle kinase Cdc28. Our phosphoproteomic analysis suggests that different harvesting methods can induce specific stresses in a short time. In fact, we observed that filtration may increase cell wall stress and the osmotic stress that does not occur during centrifugation. Thus, in some cases, an analysis of proteome stability must be accompanied by an analysis of posttranslational modification (such as phosphoproteomic analysis) to take account of the complexity of the proteome and differences in proteoforms. 

In summary, our study suggests the importance of taking into consideration the fact that certain harvesting conditions affect the basal activation levels of specific stresses. In our study, only the behavior of wild type cells in the exponential growth phase have been examined. It follows that other yeast strains could behave differently than wild type. For example, yeast mutants that are defective in these stress pathways could be more sensitive than wild type to specific harvesting methods, a hypothesis which requires further testing. It is also possible that growth conditions could affect how yeast cells respond to different harvesting methods. For example, cells in the stationary growth phase, that are known to be more resistant to stress, could be less sensitive to the harvesting method used. Such experiments, that use the strategies and techniques outlined herein, can expand further our findings to other strains and conditions. Moreover, the stress response activation that we observed is likely transient and cells should revert to their normal state over time. Further studies will be needed to better delineate the kinetics and the temporal window of the stress response and to what degree the cellular proteome and phosphoproteome revert to their normal state. Thus, the choice of harvesting method should be carefully considered when designing experiments. 

## Data Availability

RAW files have been deposited to the ProteomeXchange Consortium via the PRIDE [21] partner repository with the dataset identifier PXD044387. Reviewer account details: Username: reviewer_pxd044387@ebi.ac.uk and Password: 0UjdfC8f. The data are freely available.

## Supplementary Materials

The following supporting information can be downloaded at: https://www.mdpi.com/article/10.3390/proteomes11040028/s1, Figure S1: Global phosphoproteome analysis and examples of phosphorylation events increasing after filtration. Table S1: List of the proteins, list of the peptides with their relative abundances used for quantification and list of the phosphosites with their relative abundances.
