# Supplementary material for: Comparison of the Proteomes and Phosphoproteomes of S. cerevisiae Cells Harvested with Different Strategies
Source: Proteomes. 2023 Sep 27;11(4):28. doi: 10.3390/proteomes11040028 (PMC10594529; doi:10.3390/proteomes11040028)

(A)

Conditions	Phosphorylation events increasing	Phosphorylation events decreasing
Hi./Lo. speed	18	13
Hi speed/filtration	100	175
Lo. speed/filtration	42	103

log_2Fc 0.75
p-value<0.05

(B)

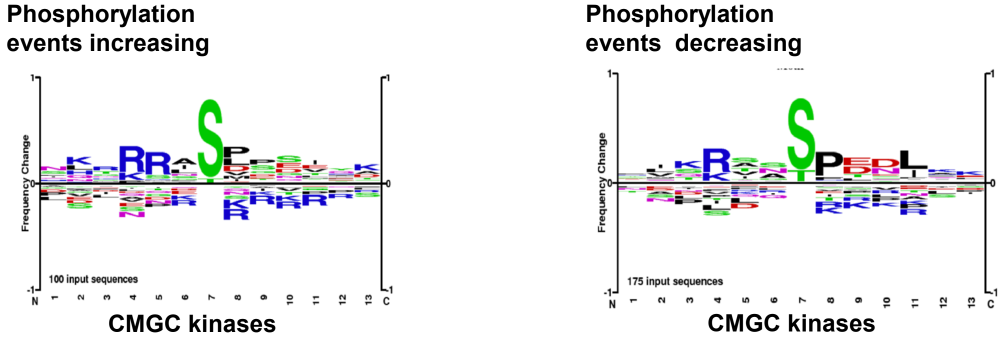

(C)

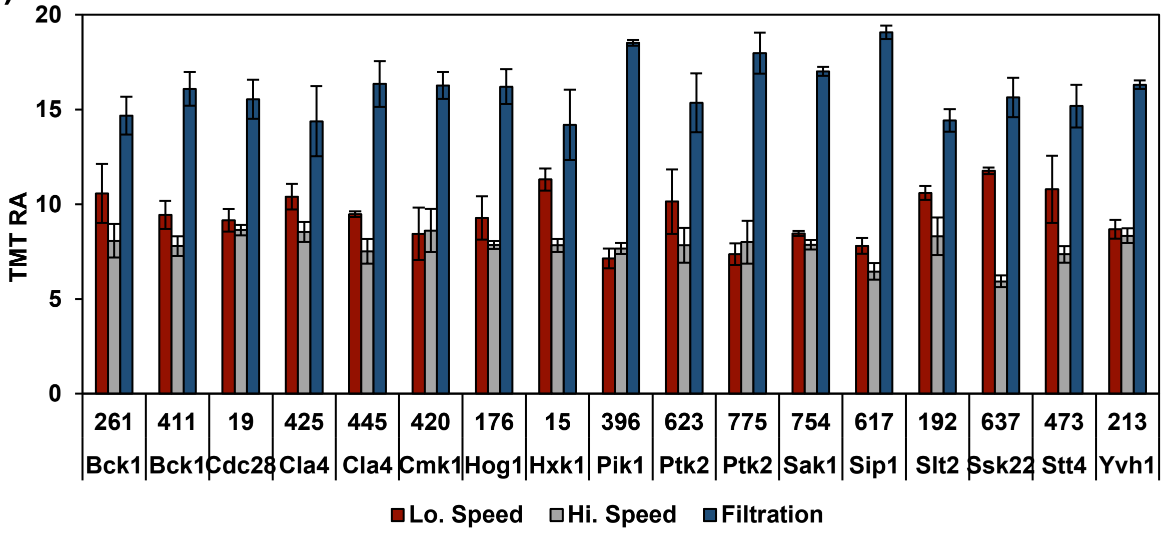

(D)

Kinase inactivation: Phosphorylation outside of the activation loop

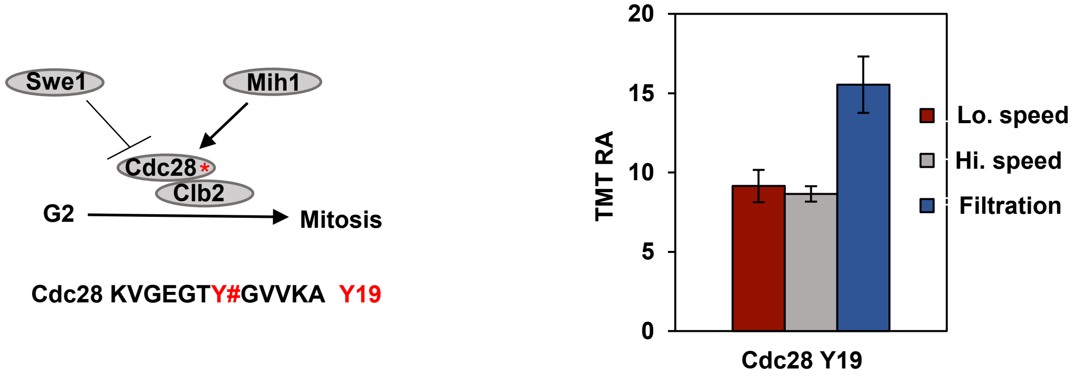

Supplement: Supplementary file 1 [file proteomes-11-00028-s001.zip › proteomes-2572280-supplementary-figure s1_updated.pdf]
